# Study on Microstructure and Stress Distribution of Laser-GTA Narrow Gap Welding Joint of Ti-6Al-4V Titanium Alloy in Medium Plate

**DOI:** 10.3390/ma18132937

**Published:** 2025-06-21

**Authors:** Zhigang Cheng, Qiang Lang, Zhaodong Zhang, Gang Song, Liming Liu

**Affiliations:** 1 School of Materials Science and Engineering, Dalian University of Technology, Dalian 116024, China; cheng03252022@163.com (Z.C.); skyezzd@dlut.edu.cn (Z.Z.); liulm@dlut.edu.cn (L.L.); 2 Key Laboratory of Liaoning Advanced Welding and Joining Technology, Dalian University of Technology, Dalian 116024, China; 3 School of Mechanical Engineering, Dalian University of Technology, Dalian 116000, China; lqiang_dlut@163.com; 4 Liaoning Huanghai Laboratory, Dalian University of Technology, Dalian 116000, China

**Keywords:** laser-induced arc flexible heat source, thick Ti-alloy plate, ultrasonic impact microstructure, impact toughness, residual stress

## Abstract

Traditional narrow gap welding of thick titanium alloy plates easily produces dynamic molten pool flow instability, poor sidewall fusion, and excessive residual stress after welding, which leads to defects such as pores, cracks, and large welding deformations. In view of the above problems, this study takes 16-mm-thick TC4 titanium alloy as the research object, uses low-power pulsed laser-GTA flexible heat source welding technology, and uses the flexible regulation of space between the laser, arc, and wire to promote good fusion of the molten pool and side wall metal. By implementing instant ultrasonic impact treatment on the weld surface, the residual stress of the welded specimen is controlled within a certain range to reduce deformation after welding. The results show that the new welding process makes the joint stable, the side wall is well fused, and there are no defects such as pores and cracks. The weld zone is composed of a large number of α′ martensites interlaced with each other to form a basketweave structure. The tensile fracture of the joint occurs at the base metal. The joint tensile strength is 870 MPa, and the elongation after fracture can reach 17.1%, which is 92.4% of that of the base metal. The impact toughness at the weld is 35 J/cm^2^, reaching 81.8% of that of the base metal. After applying ultrasound, the average residual stress decreased by 96% and the peak residual stress decreased by 94.8% within 10 mm from the weld toe. The average residual stress decreased by 95% and the peak residual stress decreased by 95.5% within 10 mm from the weld root. The residual stress on the surface of the whole welded test plate could be controlled within 200 MPa. Finally, a high-performance thick Ti-alloy plate welded joint with good forming and low residual stress was obtained.

## 1. Introduction

Titanium alloys (Ti-alloys) exhibit high specific strength [1], excellent corrosion resistance, superior weldability, and non-magnetic properties. These alloys find extensive applications in biomedical, aerospace, and deep-sea engineering applications [2,3]. Notably, the demand for thick titanium alloy welded structures in the field of marine engineering has surged by 30% [4,5]. The development of high-efficiency, high-performance welding techniques for thick-section titanium alloys has emerged as a critical challenge requiring immediate attention in key national strategic sectors [6].

At present, the main welding method of thick Ti-alloy plates is narrow gap welding with a single heat source [7], such as GTAW [8], laser welding [9], or vacuum electron beam welding [10]. Feng et al. [8] successfully welded 20-mm-thick TC4 Ti-alloy plates using the TIP-TIG welding method. Microstructural analysis revealed that (heat-affected zone) HAZ exhibited a typical bimodal structure comprising equiaxed α-, β-, and α′-phases, with an average microhardness of 350 HV. The weld zone (WZ) contained a basketweave microstructure of α- and β-phases, demonstrating an average microhardness of 315 HV. The welded joint exhibited an average tensile strength of 1043 MPa, corresponding to approximately 90% of the base material’s strength. The calculated specific strength was 23.2 N m/kg. André et al. [9] used a 20 kW laser to weld 16-mm-thick titanium alloy plates. The microstructure of the weld was acicular α′ martensite, and the weld penetration was difficult to exceed 20 mm. Sun et al. [10] achieved high-quality joining of 20-mm-thick TC4 Ti-alloy plates by vacuum electron beam welding. The results showed that the grain size decreased gradually along the depth direction, the grain size of the fusion zone was the smallest, and the tensile strength was the highest [11,12]. The tensile results showed that the tensile strength could reach 95% of that of the base metal. The tensile results showed that the tensile strength was 980 MPa. The calculated specific strength was 21.7 N m/kg. Although vacuum electron beam welding has high energy density, strong penetration ability, and high efficiency [13], this method has high requirements for the size of welded components and harsh welding conditions [14]. Research shows that the hybrid heat sources have stronger applicability, a higher energy utilization rate, smaller welding heat input, and higher energy density [15,16].

The laser-GTA flexible hybrid heat source has a stable arc and high energy utilization rate, which is suitable for the welding of thick TC4 Ti-alloy plates [17]. During the welding process, a large amount of residual stress is generated at the weld due to unbalanced cooling, which affects the safety of welded components [18]. According to the standard ‘Structural Welding Code-Stainless steel’ [19], the residual stress should be controlled to be less than 0.3 times the yield strength to ensure that the welded components are not deformed and not cracked, and to ensure the integrity and safety of the structure. However, the distribution and elimination of residual stress in laser-GTA hybrid welding of thick Ti-alloy plates have not been solved. In recent years, many scholars have studied the distribution law of welded residual stress of Ti-alloys. Liu et al. [20] studied the microstructure and residual stress distribution of 7.5-mm-thick Ti-alloy plates through experiments and the thermal elastic-plastic finite element method. The results showed that the columnar crystals in the lower part of the molten pool were smaller than those in the upper part of the molten pool, and the grain growth direction of the welded joint was consistent with the simulation results. The stress field showed that there was a high transverse tensile stress near the interlayer. There was a stress concentration phenomenon in the weld toe and weld root [21], which made it difficult to meet actual production needs. Studies have shown that ultrasonic impact treatment can effectively remove the residual stress inside the welded specimen and improve the mechanical properties of the welded joint [22]. Xu et al. [23] studied the effect of ultrasonic treatment on the properties of TC4 Ti-alloy laser welded joints. The results showed that a plastic deformation layer was formed on the surface of the welded joint after ultrasonic impact treatment, and the fatigue strength was increased by 4.91%.

Previous experiments have shown that the laser-GTA flexible hybrid heat source has good stability, and side wall fusion is promoted by the laser heat source-induced arc heat source stable discharge [24], but the welded residual stress is large, which affects the welded joint properties. In the measurement of residual stress, the X-ray diffraction method is widely used because of its high precision and wide application range [25]. On the basis of this experiment, the residual stress of the welded sample was measured using the X-ray diffraction method, and the distribution law was studied. The welded residual stress generated was removed by instant ultrasonic impact treatment, and the welded residual stress was controlled within a certain range.

This study employed 16-mm-thick TC4 Ti-alloy plates as the base material with 1.6-mm-diameter TC4 filler wire. Utilizing pre-optimized parameters [24], we implemented a novel low-power pulsed laser-GTA hybrid welding process with synchronous ultrasonic impact treatment, successfully producing narrow gap joints exhibiting excellent formability, a defect-free microstructure, reduced residual stress (<200 MPa), and enhanced mechanical properties. The microstructure, mechanical properties, residual stress distribution of the welded joint, and the effect of ultrasonic impact on the residual stress of the welded joint were studied. The research results of this paper are of great significance to the optimization of process parameters and residual stress evaluation of laser-GTA flexible heat source welding.

## 2. Materials and Test Methods

**Figure 1:**
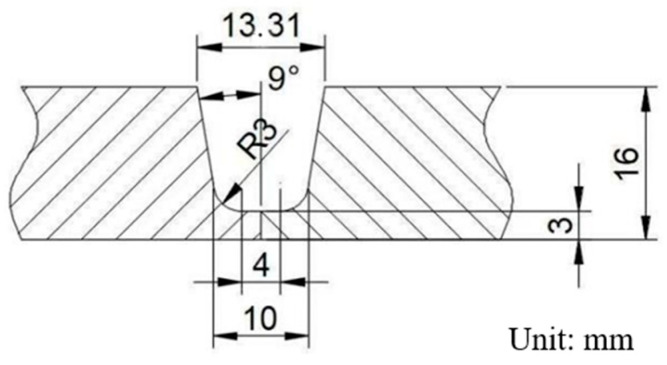
Diagram of welding groove.

In this paper, the annealed TC4 titanium alloy plate produced by Western Titanium Technology Co., Ltd. (Xi’an, China) is used as the experimental material, and its size is 300 mm (length) × 100 mm (width) × 16 mm (thickness). The welding area adopts a U-shaped groove design, and the groove size is shown in Figure 1. Welding is carried out by butt welding. The TC4 titanium alloy welding wire with a diameter of 1.6 mm is used as the filler metal. The composition of the welding wire is the same as that of the base metal. The tungsten electrode diameter is 3.2 mm. The chemical compositions of the base metal and the welding wire are shown in Table 1, and the mechanical properties of the base metal are shown in Table 2.

**Figure 2:**
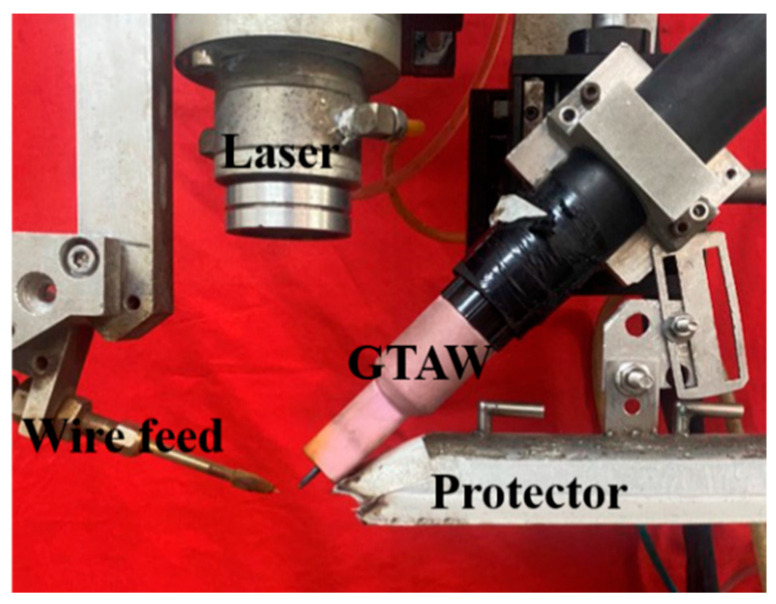
Flexible heat source welding device.

The experiments use a low-power pulsed laser-induced arc hybrid welding system, which consists of a laser optical system, an arc welding system, a motion control system, and a wire feeding system. The laser optical system is mainly composed of a low-power pulsed Nd: YAG solid-state laser (TruPulse556, TRUMPF SE + Co. KG., Ditzingen, Germany) with a maximum power of 1 kW, and the laser beam wavelength is 1064 nm. The arc welding system is mainly composed of an OTC AEP-500 P TIG welding machine (OTC Industrial (Shanghai) Co., Ltd., Shanghai, China) with a peak current of 500 A. The arc welding torch is fixed on the laser shell through a self-made welding fixture, as shown in Figure 2. The motion control system consists of a two-dimensional motion slide composed of Mitsubishi motors and a computer-integrated CNC control programme to control the direction, length, and speed of the weld, while the wire feed system consists of a GTA wire feeder (DAIHEN HC-71D, OTC Industrial (Shanghai) Co., Ltd., Shanghai, China) with a maximum wire feed speed of 5000 mm/min.

**Figure 3:**
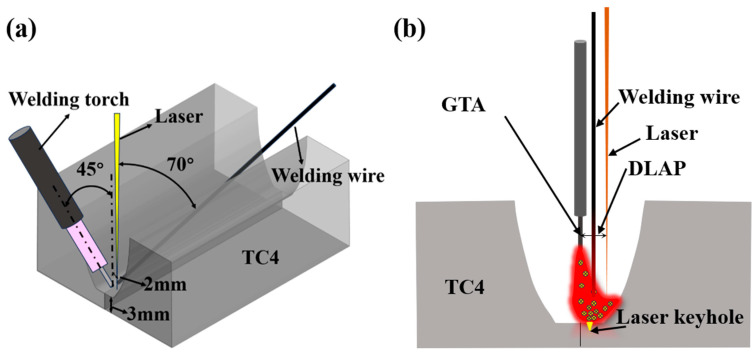
Schematic diagrams of (**a**) spatial position arrangement and (**b**) cross-section of welding process.

Figure 3 presents a schematic diagram of the welding system. Figure 3a shows the spatial arrangement of the laser, arc, and wire in narrow gap welding. Figure 3b shows the cross-section diagram of the welding process. During welding, the direction of the laser beam is perpendicular to the weld. The angle between the welding wire and the sample is 20°. The laser is perpendicular to the weld at 1.5 mm from the left side wall of the weld. The angle between the tungsten electrode and the welded sample is 45°. The lateral offset distance (DLAP) between the tungsten electrode and the laser is 2 mm. The welding wire is placed between the laser and the tungsten electrode. According to previous tests, the low-power pulse laser is used in welding. The wavelength of the laser is 1064 nm, the minimum spot diameter is 0.6 mm, the laser power is 400 W, the laser frequency is 30 HZ, the defocusing amount is 2 mm, and the pulse width is 2.5 m s. The optimized welding parameters are shown in Table 3. The welding voltage is 12 V. The gas flow rate is 15 L/min.

During the welding process, high purity argon gas (purity: 99.99%) is used to protect the front and back of the welded joint. The front of the welded joint is protected by a special protective cover, and the back of the welded joint is protected by a special protective copper tube. The whole welded joint is completed through three processes of backing welding, filling welding, and cover welding.

**Figure 4:**
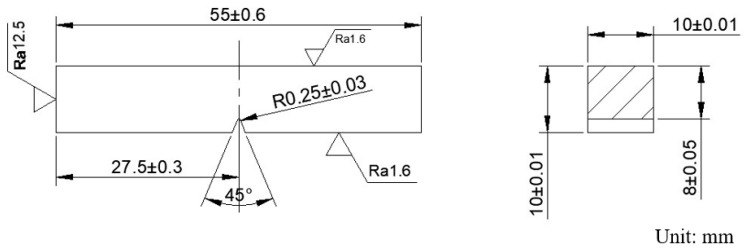
Size of the impact specimen.

**Figure 5:**
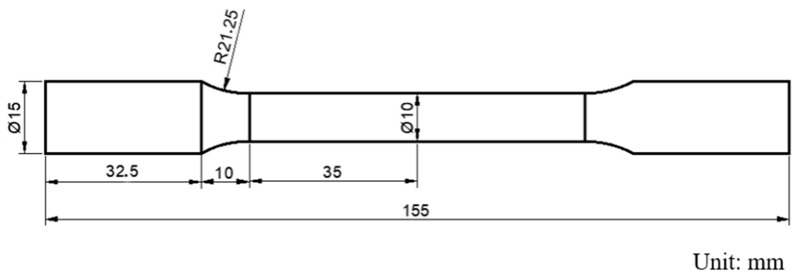
Size of the tensile specimen.

**Figure 6:**
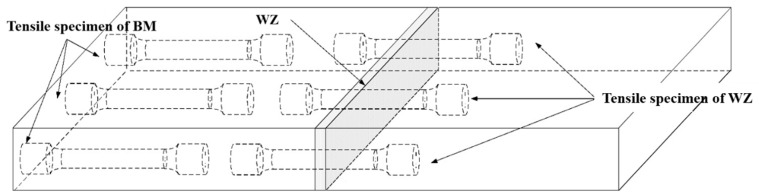
Stretching sampling position.

The central part of the welded sample is cross-cut, ground, and polished. The cross-section of the polished sample weld is immersed in a mixed solution of 6 mL HF + 12 mL HNO_3_ + 82 mL H_2_O for 20 s. An optical microscope (OM, MEF-3, Leica Camera Trading (Shanghai) Co., Ltd., Shanghai, China) and a field emission scanning electron microscope (SEM, JSM-IT800, JEOL Ltd., Beijing, China) are used to observe the microstructure of the base material zone (BM), weld zone (WZ), and heat-affected zone (HAZ). The Touch HV-1000A microhardness tester (Shenzhen Senyu Instrument Equipment Co., Ltd., Shenzhen, China) is used to test the microhardness of the weld cross-section. The applied load is 1000 gf, the indentation time is 15 s, and the distance between adjacent test points is 0.5 mm. The size of the standard impact specimen is shown in Figure 4. The impact sampling position is in the middle of the weld. The size of the standard tensile specimen is shown in Figure 5, and the tensile sampling position is shown in Figure 6. The tensile test at room temperature is carried out at a rate of 3 mm/min using SDS100 electronic universal testing machine (Wenzhou Sundoo Instrument, Co., Ltd., Wenzhou, China). The room temperature impact test is carried out using a JB-300 B impact tester (Fang Yuan Testing Machine, Jinan, China).

**Figure 7:**
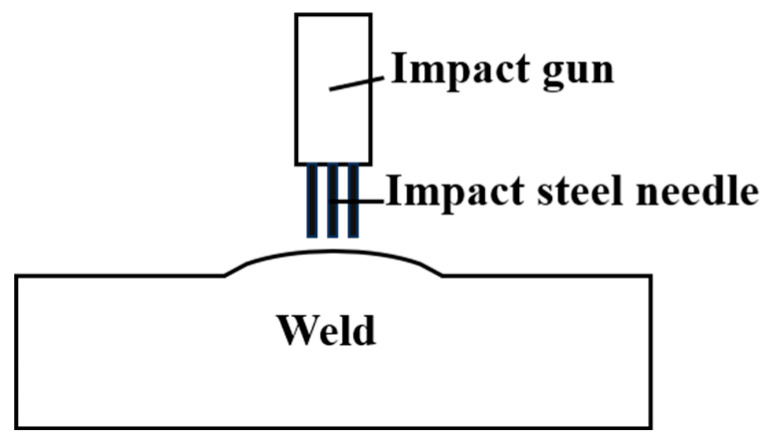
Diagram of ultrasonic impact.

In order to solve the problem of excessive welded residual stresses, post-ultrasonic treatment (UIT) is performed using the SH20000 Ultrasonic Impact Tester (Q-Z Mechanical, Shanghai, China) in the center of the weld within two minutes of the completion of each filler weld layer. The schematic diagram of ultrasonic impact is shown in Figure 7. The diameter of the impact head is 5 mm, and the impact needle adopts a single-row three-needle structure layout. The specific ultrasonic impact parameters are shown in Table 4. After the filling welding of each layer is completed, each layer of the weld is impacted twice in a single direction at a speed of 15 mm/min, where the impact pins are arranged perpendicular to the direction of the weld.

**Figure 8:**
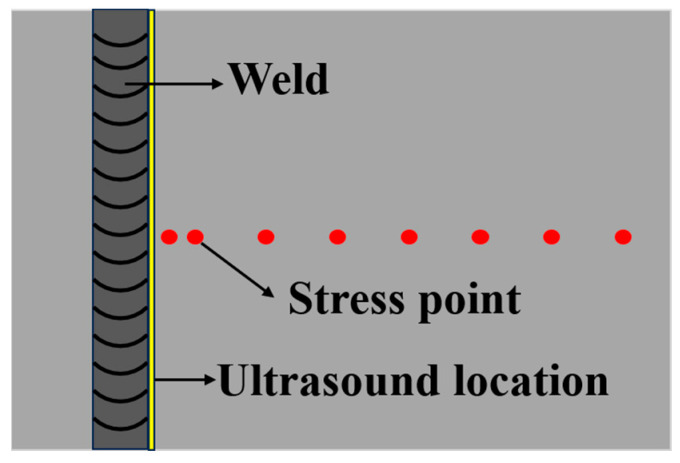
Diagram of measurement of stress point.

**Figure 9:**
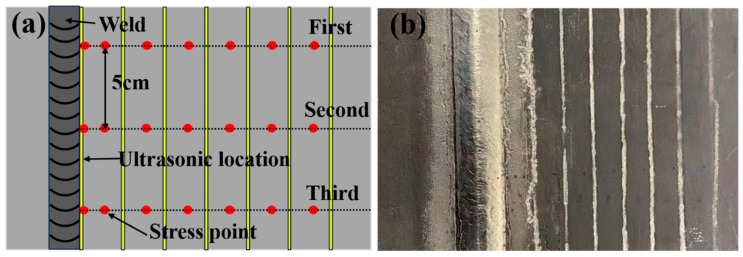
Diagrams of (**a**) measurement point of stress and (**b**) ultrasonic position.

According to the X-ray diffraction method, the residual stresses are determined around the weld in the direction perpendicular to the weld using an XL-640 residual stress tester (Handan Aiste Stress Technology Co., Ltd., Handan, China) with a radiation source of Cu, an X-ray tube pressure of 26 kV, a tube current of 6 mA, and a diffraction plane of {213}. The method of fixing the peaks is used in the Cauchy method, and the range of the 2θ angle is 147° for the high angle and 136° for the low angle. In order to ensure the stability of the value, three measurements are carried out at three different positions at 1 mm on the left and right sides of the same point of the sample after UIT, and the average value is the residual stress value of the point. The residual stress is measured at 3 mm, 6 mm, 16 mm, 26 mm, 36 mm, 46 mm, 56 mm, 66 mm, and 76 mm from the weld toe on the sample treated by instant ultrasonic during the welding process. The sampling position of residual stress is shown in Figure 8. On this basis, in order to further study the scope and effect of ultrasound, ultrasonic impact treatment is performed every 10 mm on the front of the welded test plate. The residual stress values are measured at 5 mm, 15 mm, 25 mm, 35 mm, 45 mm, 55 mm, 65 mm, and 75 mm from the weld toe, and three groups are made at an interval of 100 mm perpendicular to the weld direction to ensure the reliability of the data. The measurement position and ultrasonic position are shown in Figure 9.

## 3. Results and Discussion

### 3.1. Macroscopic Morphology and Microstructure

**Figure 10:**
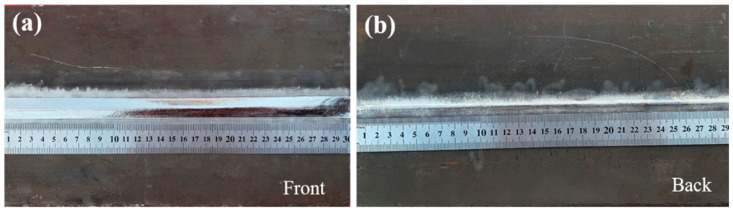
Macroscopic morphology of weld: (**a**) weld front; (**b**) weld back.

**Figure 11:**
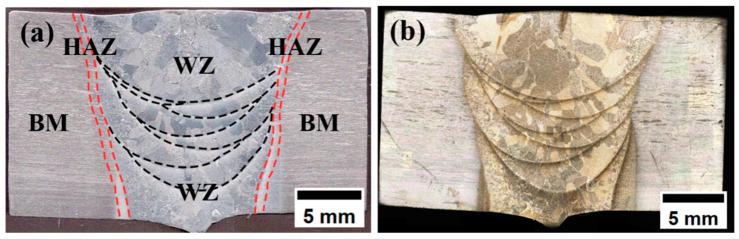
Cross-section of welded joints: (**a**) welding zone division; (**b**) cross-sectional morphology.

Figure 10 presents the macroscopic morphology of the welded specimen, exhibiting a characteristic bright silver-white appearance indicative of effective argon shielding, with no observable oxidation or welding defects (e.g., porosity or cracks). As shown in Figure 11, the joint cross-section reveals three distinct regions: the base metal (BM), weld zone (WZ), and heat-affected zone (HAZ). The micrograph demonstrates complete side wall fusion with no detectable defects, including porosity, lack of fusion, or cracking. The weld exhibits a trapezoidal profile with an upper width of 18 mm and lower width of 11 mm, featuring an exceptionally narrow HAZ with a width of approximately 0.5 mm. The consistent silver-white metallic luster on both upper and lower surfaces confirms optimal shielding gas protection during welding, preventing any titanium oxidation. The laser-arc hybrid heat source demonstrates three key advantages: (1) laser-induced arc deflection enhances side wall fusion, (2) concentrated energy density minimizes HAZ width, and (3) improved energy efficiency enables superior weld formation at reduced heat input (≤1.2 kJ/mm). These synergistic effects significantly reduce residual stresses while ensuring complete interfacial fusion [24].

**Figure 12:**
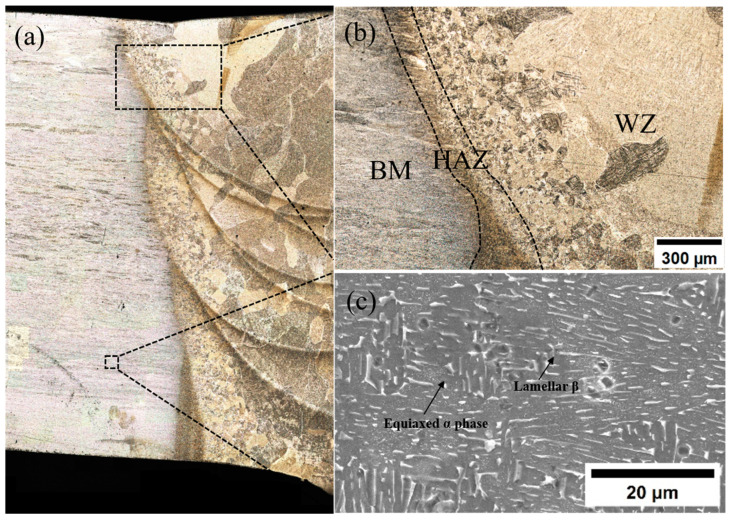
(**a**) Cross-sectional morphology of welded joints; (**b**) transition diagram of welded joints in various regions; (**c**) BM microstructure.

The overall microstructure morphology of the welded joint is shown in Figure 12. Figure 12b shows the overall morphology between the regions of the welded joint under low magnification, and it can be observed that the boundaries of the regions are clearly visible, the microstructure transition between BM and WM is relatively smooth, and the grain size gradually increases from HAZ to WZ. Figure 12c shows the morphology of the TC4 base material. It can be seen that there are equiaxed α-phase and β-phase in the microstructure of the TC4 titanium alloy. The β-grain size is small, the original β-grain boundary is not obvious, and the β-phase is dependent on the matrix α-phase, which is uniformly distributed.

**Figure 13:**
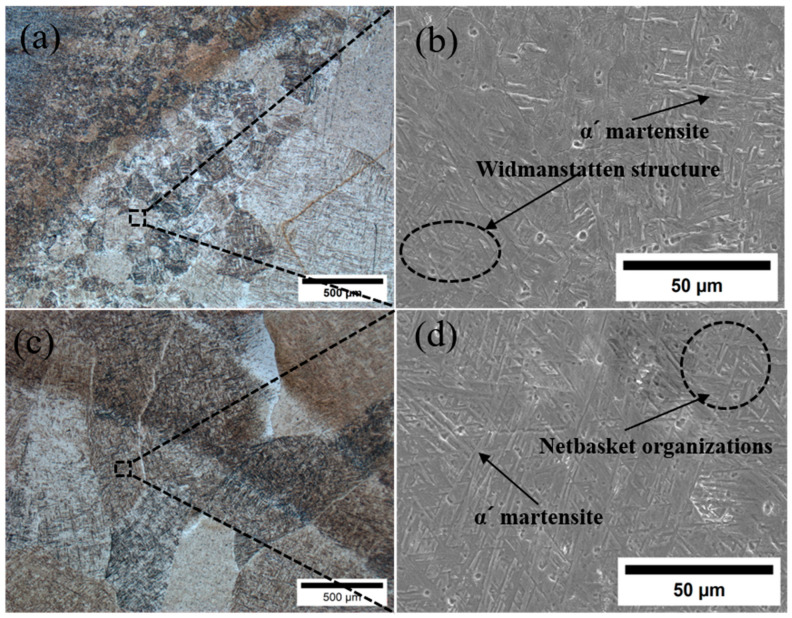
(**a**) HAZ metallographic diagram; (**b**) HAZ scanning diagram; (**c**) WZ metallographic diagram; (**d**) WZ scanning diagram.

Figure 13 shows the microstructure and morphology of HAZ and WZ. Figure 13a,b show the microstructure morphology of HAZ, and it can be seen that the grain size of HAZ is smaller and the organization is more complex. The HAZ structure near the matrix is similar to that of the matrix, which is mainly composed of equiaxed lamellar α-phase and β-phase attached to the periphery of the matrix α-phase. The middle of HAZ is composed of acicular α′ martensite and β-phase, and acicular α′ martensite accounts for the majority. Because the heat dissipation during welding has a certain direction, this also leads to the obvious direction of the growth of columnar grains in the middle part of HAZ, from the WZ zone to the BM zone. The HAZ microstructure near WZ is similar to that of WZ. Compared with the middle of HAZ, the number of acicular α′ martensites increases, β-phase decreases gradually, and a small amount of widmanstatten α-phase is contained. This is because when the temperature exceeds the β-phase transition temperature during welding, the original α-phase becomes β-phase, forming a high temperature β-phase. After welding, as the cooling rate exceeds the critical cooling rate of β-phase transition, the β-phase will be transformed into acicular α′ martensite, and a small amount of widmanstatten structure α-phase is formed in some areas with slower cooling rate. Therefore, as HAZ gradually approaches WZ, acicular α′ martensite gradually increases. The grain size of HAZ is quite different. Because the thermal conductivity of titanium alloy is not good, the high-temperature residence time of HAZ near the weld is relatively long, and the grain size of some grains grows obviously, which is a large columnar crystal. The grain size near the BM region is smaller.

Figure 13c,d show the WZ microstructure, which is composed of a large number of columnar grains with obvious grain boundaries. The interior of the grains is composed of a large number of needle-like α′ martensites woven into a basket-like structure. Due to the small heat-affected zone of laser-arc hybrid heat source welding, the welding cooling rate at the weld is fast, and the needle-like α′ martensites will be precipitated inside the β grains. The α′ martensite first forms parallel primary α′ martensite. These primary α′ martensites extend to the entire grain until they reach the grain boundary and then form a series of relatively fine secondary needle-like α′ martensites. The growth stops when the grain boundary is in contact with the primary α′ martensites. A large number of α′ martensites are intertwined in the grains to form a large number of basketweave structures. This makes the final morphology of the weld microstructure a woven structure.

### 3.2. Tensile Strength and Microhardness

**Figure 14:**
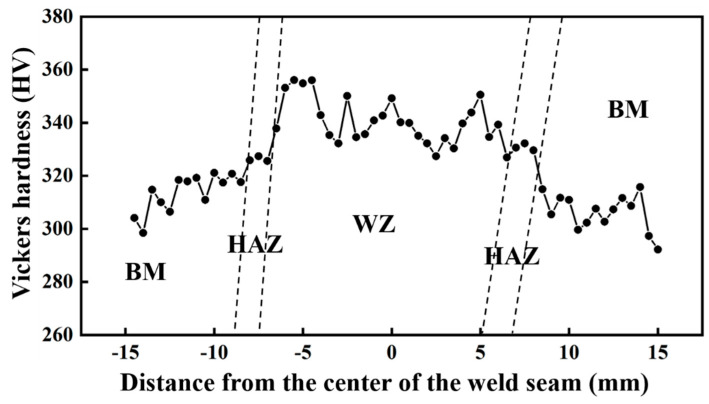
Microhardness of welded joint.

The microhardness distribution of different regions of the welded joint under this parameter is shown in Figure 14. It can be seen that the highest microhardness of the welded joint is WZ, followed by HAZ, and the lowest is BM. The average microhardness of the weld is the highest, up to 340 HV_0.2_. The peak value appears in the fusion zone near the fusion line, up to 360 HV_0.2_. The reason for the higher microhardness of the WZ and the HAZ zones is that phase transformation strengthening occurs during the welding process, and a small needle-like α′ martensite phase is generated. The α′ martensite phase generated at a faster cooling rate is a supersaturated solid solution, and there are a large number of dislocations and twins inside. These defects can effectively prevent the movement of dislocations, thereby improving the hardness of the material. More α′ martensite in WZ leads to the highest average microhardness. In the process of the welding thermal cycle, the content of β-phase increases due to local high temperature, and the grain boundary of β-phase increases, which hinders the movement of dislocation. At the same time, there is a large amount of element segregation at the grain boundary, which makes the peak value of the microhardness of WZ appear in the fusion zone near the weld [13].

### 3.3. Tensile Strength

**Figure 15:**
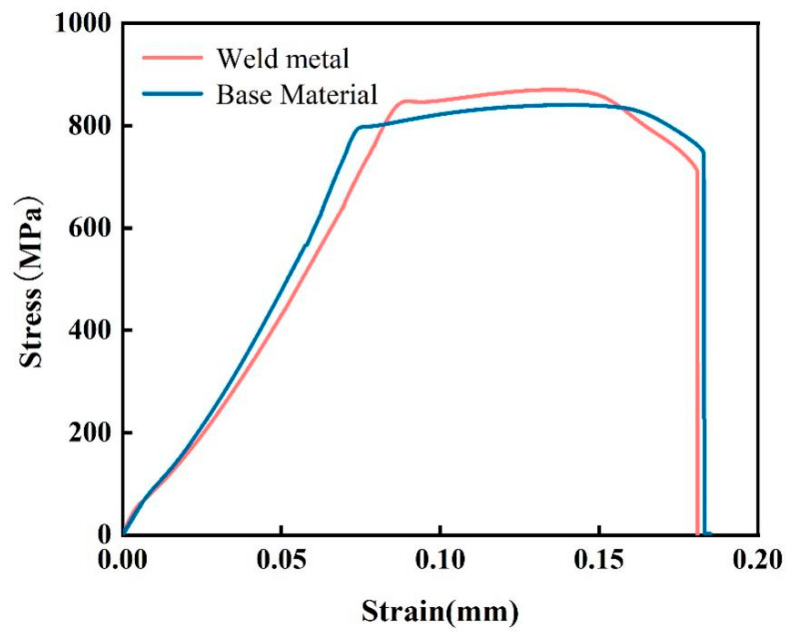
Strain–stress curve.

The static load tensile tests of the TC4 Ti-alloy base metal and welded joints are carried out at room temperature. The fracture position of the welded specimen is near BM of HAZ. The tensile curve is shown in Figure 15. The tensile strength is shown in Table 5. The tensile strength of the base metal is 840 MPa and the elongation is 18.5%. The tensile strength of the welded joint is 870 MPa, and the specific strength is 19.3 N m/kg, which is 3.58% higher than that of the base metal, and the elongation is 17.1%, which reaches 92.4% of that of the base metal. The elongation reaches 95% of that of the solution aging heat treatment, and the specific strength reaches 83% of that of the solution aging heat treatment [1]. The results show that the tensile strength of the welded joint is significantly higher than that of the base metal, which is caused by the formation of more fine α′ martensite phase at WZ and HAZ during the welding thermal cycle. It can be seen from Figure 13 that the microstructure of WZ is composed of acicular α′ martensite phase. The microstructure near WZ in HAZ is α′ martensite phase and a small amount of α-phase. The microstructure away from WZ is composed of primary α-phase, intergranular β-phase, and a small amount of α′ martensite phase. The α′ martensite phase is a supersaturated solid solution with extremely high strength and toughness. There are a large number of dislocations and twins inside it, which can effectively prevent the movement of dislocations and improve the strength of the joint [26]. Due to the effect of phase transformation strengthening, the strength and hardness of WZ and HAZ are higher than those of the base metal, and the tensile elongation is slightly lower than that of the base metal.

**Figure 16:**
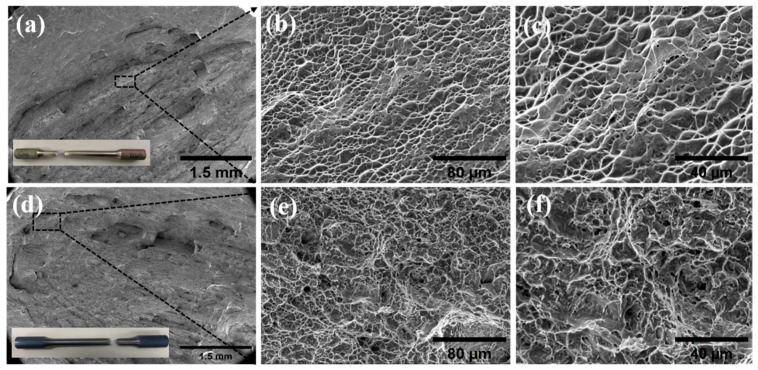
Fracture surface morphology: (**a**) low-magnification fracture surface morphology of the base metal (**b**), (**c**) enlarged image shown in the box of the (**a**), (**d**) low-magnification fracture surface morphology of the welded joint, (**e**,**f**) enlarged image shown in the box of the (**d**).

The tensile fracture morphology of the welded joints is shown in Figure 16. From the macroscopic fracture morphology, the fracture has an obvious fibrous area, the fracture surface is relatively rough, and there is obvious necking around the fracture. There are no pores, slag inclusions, and other defects at the edge of the fracture. Under SEM, it can be seen that a large number of uniformly distributed dimples are formed at the fracture. The fracture mode of the tensile sample is ductile fracture, and no obvious welding defects appear during the welding process. Figure 16b shows that the dimples formed by stretching are equiaxed dimples, indicating that the stress applied during the stretching process is uniform stress. The average size of the dimples is concentrated at about 10 μm, and the dimples are larger, indicating that the plasticity and toughness of the welded joints are better. The depth of the dimples is also deeper, indicating that the anisotropy of the material is relatively small, the internal structure is uniform, and the plasticity and toughness of the material in all directions inside the material are better [27].

### 3.4. Impact Toughness

**Figure 17:**
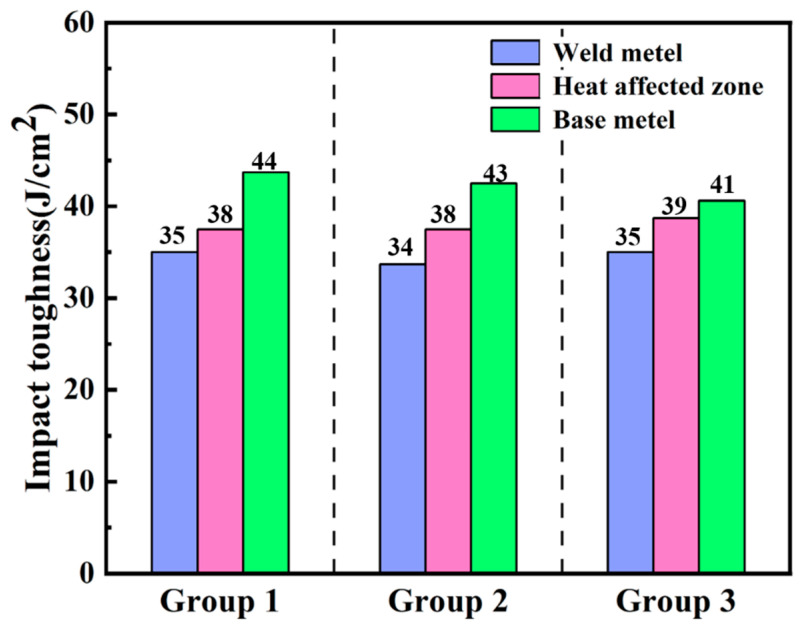
Columnar diagram of impact toughness.

The Charpy impact toughness test results are shown in Figure 17. The average impact toughness of the base metal in the three groups is 43 J/cm^2^, the impact toughness of the weld is 35 J/cm^2^, reaching 81.8% of that of the base metal, and the impact toughness of the heat affected zone is 38 J/cm^2^, reaching 89.7% of that of the base metal. The impact toughness of HAZ is greater than that of WZ. The impact toughness of the TC4 joint after oscillating laser welding is only 12 J/cm^2^ [26]. By contrast, the welded joint under the low-power pulsed laser-GTA flexible heat source welding method has better impact toughness. The decrease in the average impact toughness of the welded joint is due to the existence of a large number of unevenly distributed needle-like α′ martensites at the welded joint. It can be seen from Figure 13 that the base metal area is composed of a large number of equiaxed α-phases and β-phases attached to the α-phase. The equiaxed α-phase can effectively disperse the stress during impact, and the impact toughness is large [28].

The structure of HAZ is more complicated. There is a needle-like widmanstatten structure α-phase near the base metal, and there is needle-like α′ martensite near the weld. Due to the uneven distribution of needle-like α-phase, it easily becomes a stress concentration point during impact, which reduces the impact toughness of HAZ. At the same time, the existence of a large number of twins in the needle-like α′ martensite phase will also reduce the plastic deformation ability of HAZ, resulting in a decrease in impact toughness. There is also a small amount of untransformed β-phase in HAZ. The β-phase has good plasticity, and the grain size of HAZ is small. Compared with the number of grain boundaries in the WZ zone, more grain boundaries will hinder crack propagation, which is beneficial to improving impact toughness. Compared with the WZ zone, the impact toughness of HAZ is higher than that of the weld zone due to the existence of a small amount of untransformed β-phase and a large number of grain boundaries. At the same time, the impact toughness of HAZ is lower than that of the base metal due to the existence of acicular widmanstatten structure α-phase and α′ martensite. The weld zone is composed of a large number of interlaced acicular α′ martensites, the grain size is large, the grain boundary is less, and the impact toughness is the lowest at the whole welded joint.

**Figure 18:**
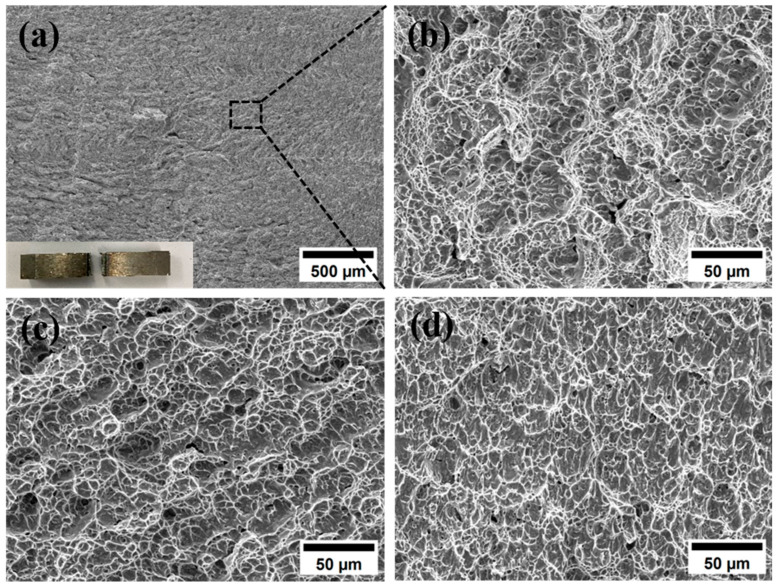
Impact fracture morphology: (**a**) WZ; (**b**) amplified view of (**a**); (**c**) BM; (**d**) HAZ.

The impact fracture morphology is shown in Figure 18. It can be seen from the fracture that the impact fracture is composed of a large number of tearing dimples. The fracture modes of the base metal, WZ, and HAZ are all ductile fracture. They are subjected to shear stress during the fracture process. The micropores inside the material will deform and expand under the action of shear stress to form tearing dimples. The tearing dimples are elongated, and the elongation direction is consistent with the shear stress direction. Compared with Figure 18c,d, the impact fracture dimples at the weld are smaller and unevenly distributed. The impact toughness at the weld is relatively poor due to the presence of a large amount of α′ martensite, and there is a certain residual stress inside the weld. Compared with the weld, the grain size of HAZ is relatively small, and its toughness is also between the base metal and the weld. In order to ensure the impact toughness of the weld, the residual stress at the weld must be eliminated. On this basis, the ultrasonic treatment of the weld is carried out to eliminate the welded residual stress and obtain a better welded joint.

### 3.5. Residual Stress Distribution Before and After Ultrasonic Impact Treatment

**Figure 19:**
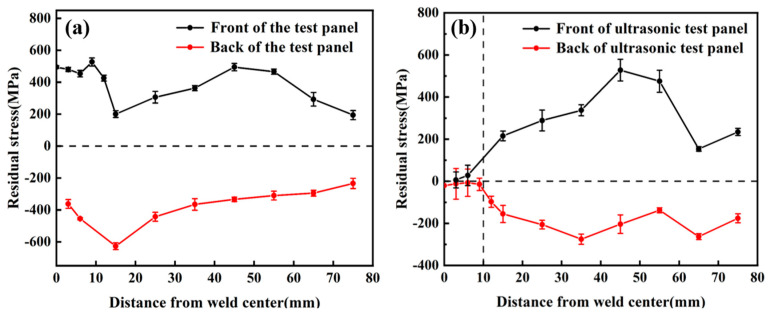
Residual stress curves: (**a**) before UIT; (**b**) after UIT.

The residual stress of the original joint and the welded joint after UIT impact treatment layer by layer is measured using the X-ray diffraction method. The residual stress results are shown in Figure 19. Figure 19a shows that the welding residual stress around the weld of the sample without UIT treatment is large. The harmful residual tensile stress at the weld toe can reach 494.8 MPa, and the average residual stress within 6 cm from the weld toe is 487 MPa. The front of the whole sample is residual tensile stress, and the residual stress of the weld toe accessory is larger. Then, with the increase in the distance from the weld toe, the residual stress increases first and then decreases. The residual stress at the weld heel reaches 362.3 MPa, and the average residual stress within 6 cm from the weld toe is 408 MPa. The residual stress on the back of the specimen is compressive stress. As the distance from the weld root increases, the residual compressive stress on the back of the welded specimen increases first and then decreases, and the average residual stress is concentrated at about 300 MPa. From a macro point of view, due to the large residual stress, the surface of the welded specimen is deformed. Due to the uneven cooling of the weld during the welding process, the cooling rate of the weld zone is faster, the cooling rate of HAZ is slower, and the inconsistency of shrinkage and expansion leads to the generation of residual stress. In addition, during the cooling process, the β-phase transforms into a needle-like α′ martensite phase, which is accompanied by volume changes and residual stress. Applying UIT after each layer of filler wire welding can effectively reduce the residual stress of the welded joint.

Figure 19b shows the residual stress distribution on the surface of the sample with immediate ultrasonic treatment during the welding process. Figure 19b shows that the average residual stress at 10 cm from the weld toe after UIT is 18 MPa, which is tensile stress. Compared with the sample without UIT treatment during the welding process, the residual tensile stress is reduced by 96%. The average residual stress at 10 mm from the weld root is 17 MPa, and the residual stress is compressive stress. Compared with the non-UIT sample, the residual stress is reduced by 95%. Compared with the sample without UIT treatment, the residual stress is greatly affected in the range of 10 mm from the weld, and the ultrasonic impact area is 10 mm. The peak value of residual stress in the area not affected by ultrasound is large and unevenly distributed. On the whole, the residual stress of the sample without UIT treatment is consistent, showing a trend of first increasing and then decreasing. During the welding process, real-time ultrasonic treatment of the weld surface solves the problem of excessive residual stress at the welded joint during the welding process of titanium alloy thick plates. When the ultrasonic wave acts on the weld surface, the high-frequency vibration of the ultrasonic wave will cause dislocation movement, grain boundary slip, and grain rotation in the sample, resulting in plastic deformation and release of residual stress due to the change of local atomic arrangement in the sample [23]. The local thermal effect of ultrasonic vibration will also release residual stress.

**Figure 20:**
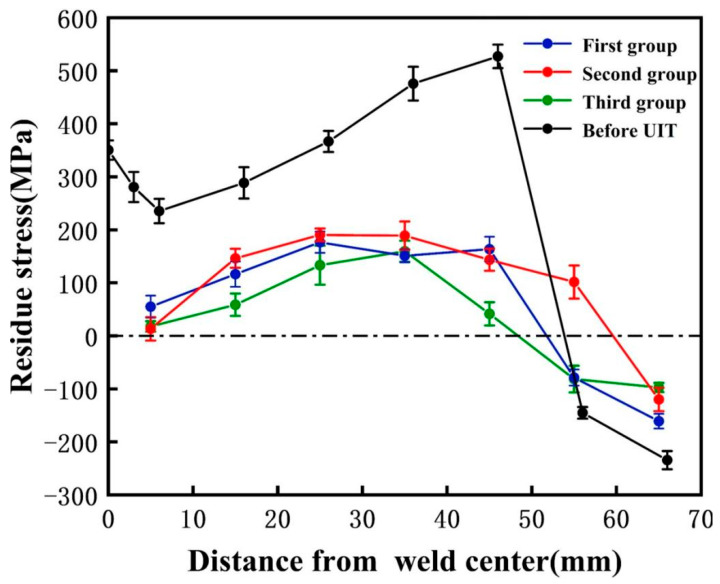
Residual stress on the surface of the test plate after UIT.

On this basis, in order to remove the residual stress on the surface of the whole sample and explore the specific scope of action of UIT, as shown in Figure 9b, UIT treatment is performed on the surface of the test plate at intervals of 100 mm from the weld toe. As shown in Figure 9a, the residual stress is measured at the middle and 5 cm left and right of the middle of the welded sample perpendicular to the weld direction. The residual stress distribution before UIT treatment is shown in Figure 20. Figure 20 shows that the peak residual stress on the surface of the test plate before UIT treatment is 527 MPa, and the average residual stress is 322 MPa. After UIT treatment, the peak residual stress decreases to 190 MPa, a decrease of 64%. The average residual stresses at three different measuring positions are 131 MPa, 141 MPa, and 83.7 MPa, respectively. The average residual stresses before and after ultrasonic treatment decrease by 59%, 56%, and 74%, respectively. The residual stresses measured at three measuring positions are all within 200 MPa. It can be seen that UIT treatment can control the residual stress of welded samples within 200 MPa, making the distribution of residual stress more uniform, and UIT treatment has a significant effect on adjusting residual stress.

In summary, in the welding process of thick Ti-alloy plates, ultrasonic treatment is applied in the process of filling the wire in each layer. Ultrasonic treatment can effectively reduce the residual stress on the surface of the welded joint during the welding process of thick Ti-alloy plates and improve the comprehensive mechanical properties of the welded joint. In addition, the distribution of residual stress outside the ultrasonic impact area remains almost unchanged, which means that the ultrasonic impact has little effect on the change in residual stress outside the impact area, and the effective ultrasonic action area is within 10 mm from the ultrasonic center. Therefore, it can be inferred that UIT can improve the residual stress in the specified area without causing stress changes at other locations.

## 4. Conclusions

In this paper, the microstructure and mechanical properties of TC4 Ti-alloy laser-TIG multi-pass narrow gap welded joints were studied, and the effect of UIT on welded joints during welding was studied. The conclusions are as follows:

(1) High-quality 16-mm-thick TC4 Ti-alloy plate welded joints with good side wall fusion, good interlayer fusion, and no porosity, oxidation, and other defects are successfully welded using the laser-arc hybrid heat source and designing the welding torch structure through multi-layer and multi-pass.
(2) The tensile fracture position of the welded joint is at the base metal, the tensile strength is 870 MPa, and the specific strength is 19.3 Nm/kg. The elongation after fracture is 17.8%, reaching 97.2% of that of the base metal and 95% of that of the solid solution aging heat treatment. The average microhardness of the weld zone is the highest, up to 340 HV. During the welding process, α′ martensite is formed in WZ and HAZ, which enhances its strength and hardness.
(3) The uneven distribution of acicular α′ martensite in the weld reduces its toughness, resulting in the impact toughness of the weld being 35 J/cm^2^, reaching 81.8% of that of BM, and the impact toughness of HAZ is 38 J/cm^2^, reaching 89.7% of that of BM.
(4) After UIT, the average residual stress within 10 mm from the weld toe is 18 MPa, which is tensile stress, and the average residual stress at the weld root is 17 MPa, which is compressive stress. Compared with that before UIT, the residual stress at the welded joint is reduced by more than 95%. After ultrasonic impact treatment every 10 mm on the surface of the whole test plate, the residual stress on the surface of the whole welded specimen can be controlled within 200 MPa.

## Figures and Tables

**Table 1:** Chemical composition of TC4 Ti-alloy [17] (wt.%).

Element	C	O	Fe	V	Al	Ti
Content	<0.10	<0.20	<0.30	3.5~4.5	5.5~6.8	Bal

**Table 2:** Mechanical properties of base metal.

σ_b_ (MPa)	σ_n_ (MPa)	Elongation (%)
840	790	18.5

**Table 3:** Welding parameters.

Welding Manner	Current/A	Welding Speed/(mm·min^−1^)	Wire Feed Rate/(mm·min^−1^)
Backing welding	170	300	nil
Filling welding	200	150	1000
Cover welding	220	150	1200

**Table 4:** Ultrasonic impact parameter.

Output Frequency/kHz	Output Amplitude/μm	Power/W	Impact Velocity/m·s^−1^
40	100	1500	2.5

**Table 5:** Tensile strength of base metal and joint.

	Tensile Strength (MPa)	Elongation	Breaking Position
Base metal	840.3 ± 10.6	18.5%	Base metal
Joint	870.1 ± 7.5	17.1%	Base metal

## Data Availability

The original contributions presented in this study are included in the article. Further inquiries can be directed to the corresponding author.
